# Protective Effects of Liquiritigenin against Cisplatin-Induced Nephrotoxicity via NRF2/SIRT3-Mediated Improvement of Mitochondrial Function

**DOI:** 10.3390/molecules27123823

**Published:** 2022-06-14

**Authors:** Meng Zhou, Yanpeng Dai, Yong Ma, Yi Yan, Min Hua, Qi Gao, Xue Geng, Qian Zhou

**Affiliations:** 1 The Second Hospital, Cheeloo College of Medicine, Shandong University, Jinan 250033, China; zhoumeng006@163.com; 2 Shandong Academy of Chinese Medicine, Jinan 250014, China; daiyanpeng1027@163.com (Y.D.); huamlkh@163.com (M.H.); gq20211122@163.com (Q.G.); 3 Shandong Modern Research and Development Engineering Center of Traditional Chinese Medicine Aromatherapy, Jinan 250014, China; 4 National Institute of Healthcare Security, Capital Medical University, Beijing 100037, China; mayong_0517@163.com; 5 Union Hospital, Tongji Medical College, Huazhong University of Science and Technology, Wuhan 430022, China; ezhouyanyi@163.com; 6 School of Pharmacy, Shandong University of Traditional Chinese Medicine, Jinan 250355, China; 7 NMPA Key Laboratory for Research and Evaluation of Generic Drugs, Shandong Institute for Food and Drug Control, Jinan 250101, China

**Keywords:** liquiritigenin, NRF2, cisplatin, SIRT3, mitochondrial biogenesis

## Abstract

Acute kidney injury (AKI) induced by cisplatin (CP), a first-line anticancer drug for chemotherapy, is common. To date, there is an urgent need to find effective treatments to reduce the nephrotoxicity caused by CP. Meanwhile, the restoration of mitochondrial dysfunction shows potential to be used as an adjunct to conventional therapeutic strategies. This study found that liquiritigenin can ameliorate mitochondrial dysfunction and acute kidney injury induced by CP in mice. The intraperitoneal injection of 15 mg/kg body weight liquiritigenin for 2 days markedly protected against CP-induced mitochondrial dysfunction, restored renal tubule and mitochondrial morphology, decreased blood Scr and BUN levels, and decreased cell apoptosis. Furthermore, the elevated expression of SIRT3 induced by liquiritigenin, which can be upregulated by NRF2, was confirmed in vivo and in vitro. The underlying protective mechanisms of liquiritigenin in CP-induced nephrotoxicity were then investigated. Molecular docking results showed that liquiritigenin has potent binding activities to KEAP1, GSK-3β and HRD1. Further results showed that liquiritigenin induced the nuclear translocation of NRF2 and increased the levels of mitochondrial bioenergetics-related protein such as PGC-1α, and TFAM, which are related to NRF2 activity and mitochondrial biogenesis. In addition, liquiritigenin was found to possibly reverse the decrease in BCL2/BAX ratio induced by CP in live cultured renal tubule epithelial cells. Collectively, these results indicated that liquiritigenin could be used as a potential nephroprotective agent to protect against cisplatin-induced acute kidney injury in a NRF2-dependent manner by improving mitochondria function.

## 1. Introduction

Cisplatin (CP), representing first-line anticancer drugs for chemotherapy is widely used for the treatment of solid tumors such as ovarian cancer, prostate cancer, testicular cancer, and lung cancer in the clinic [1,2]. However, the side effects of CP, particularly nephrotoxicity, limit its clinical application. Therefore, there is an urgent need to find effective treatments to reduce the nephrotoxicity caused by CP. CP compromises cellular integrity by damaging nuclear DNA to induce cell death, but several lines of evidence indicate that mitochondrial DNA (mRNA) is strongly affected by CP as well [3]. Mitochondria are energy-producing organelles that generate adenosine triphosphate (ATP) and reactive oxygen species (ROS) in cells [4,5]. Impaired mitochondria induced by CP increase the generation of ROS. When ROS generation exceeds cellular ROS scavenging capacity or antioxidant safeguards, oxidative stress occurs and accelerates mitochondrial injury [6,7]. Therefore, reducing oxidative damage to mitochondria by enhancing antioxidative capability and restoration of mitochondrial dysfunction has been proposed as a potential therapeutic strategy for acute kidney disease (AKI) induced by CP in model organisms ranging from mice to humans [8,9].

Nuclear erythroid 2-related factor 2 (NRF2) is an antioxidant transcription factor, that can combine with the antioxidant response elements (AREs) in the promoters of many genes. The regulation of NRF2 activity is closely related to Kelch-like-ECH-associated protein (KEAP1), an adaptor component for the Cullin3-based E3 ubiquitin ligase complex, which is known as the canonical KEAP1-NRF2 pathway. Under oxidative stress, the cysteine residues in KEAP1 are modified to promote the release and nuclear translocation of NRF2 [10]. NRF2 can also be regulated by GSK-3β [11], which is known as the uncanonical KEAP1-NRF2 pathway. Activated NRF2 plays an important role in the restoration of mitochondrial function by modulating the transcriptional activity of NRF2 over the downstream targets [12,13]. On one hand, ROS released by damaged mitochondria can promote the expression of antioxidant enzymes through NRF2 activation to restore redox homeostasis in mitochondria. On the other hand, NRF2 can mediate the expression of mitochondrial fusion protein and proteasome genes to inhibit mitochondrial fission with the purpose of remedying mitochondrial dysfunction [14]. Mitochondrial Sirtuin 3 (SIRT3), which is essential for maintaining mitochondrial function, is widely expressed in mitochondria-rich tissues, such as kidney, heart, brain and liver tissues [15,16].

Liquiritigenin (4′,7-dihydroxyflavone) is a major bioactive ingredient extracted from the root of licorice (*Glycyrrhiza uralensis*), a medicinal plant used in China. Studies have shown that liquiritigenin has a variety of biochemical and pharmacological properties, including hepatoprotection, anti-hyperlipidemic, anti-oxidant, anti-inflammatory and anti-cancer properties [17,18]. It has been shown that the administration of licorice ameliorates CP-induced hepatotoxicity and nephrotoxicity through anti-apoptosis, anti-oxidative stress, anti-inflammation, and accelerated metabolism [19,20]. Likewise, there is convincing evidence that 18α-glycyrrhetinic acid (GA), which is another major active component of licorice, and its metabolites might act as chemoprotectants against nephrotoxicity with anti-oxidant stress and anti-inflammatory activities [21]. Liquiritigenin was found to potentiate the inhibitory effects of CP on invasion and metastasis via the downregulation of MMP-2/9 and the PI3K/AKT signaling pathway in B16F10 melanoma cells and a mice model [22]. However, the effect and underlying mechanisms of liquiritigenin against CP-induced AKI have not yet been clarified.

Thus, this study aimed to investigate the protective effect of liquiritigenin on CP-induced AKI by improving mitochondrial function in tubular epithelial cells and mice, and to discuss the possible mechanisms. Results showed that, liquiritigenin (i) plays a role in the protection of nephrotoxicity induced by CP; (ii) has potent binding activities to KEAP1, GSK-3β and HRD1 and can induce nuclear translocation of NRF2; (iii) reverses CP-induced decreased expression of SIRT3 and induces up-regulated protein levels of peroxisome proliferator-activated receptor-γ co-activator-1α (PGC-1α) and mitochondrial transcription factor A (TFAM), which can improve the mitochondrial bioenergetics and kidney function post-injury in SIRT3 pathway; and (iv) affects the expression levels of BCL-2/BAX and the activation of downstream cell apoptotic events. Taken together, these results shed light on the interaction between liquiritigenin and the NRF2/SIRT3 pathway in CP-induced nephrotoxicity, suggesting that targeting mitochondrial function by liquiritigenin may provide unexpected opportunities for the treatment of CP-induced AKI patients.

## 2. Results

### 2.1. Liquiritigenin Protected against CP-Induced AKI in Mice

**Figure 1:**
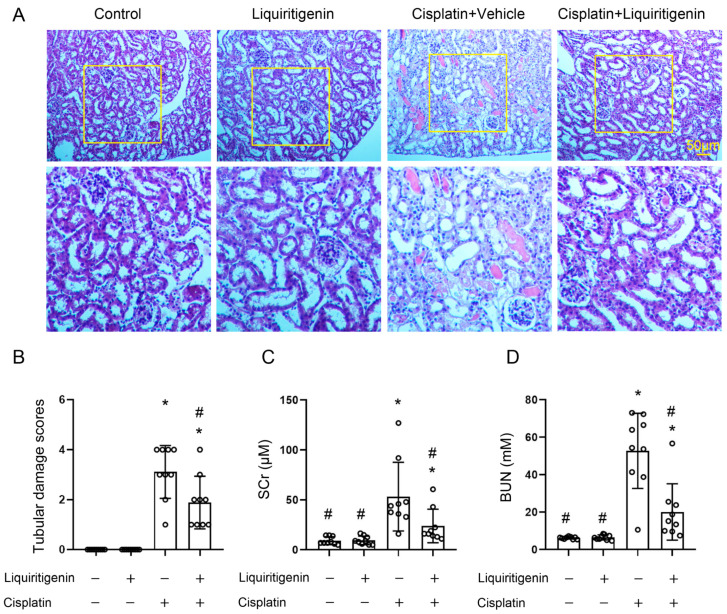
The effect of liquiritigenin on AKI induced by CP in mice. (**A**) Representative micrographs of HE staining in the kidney from different groups of mice. (**B**) Quantitative pathological assessment of tubular damage from different groups of mice. Renal morphology was scored according to the proportion of damaged renal tubules, such as brush border, tubule dilatation, and cast formation in the total renal tubules. The scoring criteria were as follows: 0, normal; 1, <10%; 2, 10~25%; 3, 26~50%; 4, 51~75% and 5, >75%. * *p* < 0.05 vs. control group of mice, # *p* < 0.05 vs. mice with CP treatment (*n* = 9 mice/group). (**C**) Serum creatinine levels of mice in different groups. * *p* < 0.05 vs. control group of mice, # *p* < 0.05 vs. mice with CP treatment (*n* = 9 mice/group). (**D**) Blood urea nitrogen levels of mice in different groups. * *p* < 0.05 vs. control group of mice, # *p* < 0.05 vs. mice with CP treatment (*n* = 9 mice/group).

As shown in Figure 1, 3 days after CP treatment, BALB/c mice displayed several morphological injuries to the kidney, as evidenced by brush border loss, tubule dilatation, and cast formation (Figure 1A), and elevated serum creatinine (SCr, Figure 1C) and blood urea nitrogen (BUN, Figure 1D). HE staining showed that liquiritigenin can obviously alleviate the change in morphology observed in the model group, with lower tubular damage scores (Figure 1A,B). Likewise, liquiritigenin significantly reduced serum creatinine and urea nitrogen levels compared to the model group, demonstrating its significant improvement effect in renal function against CP (Figure 1C,D).

### 2.2. Liquiritigenin Reduced CP-Induced Apoptosis of Renal Tubule Epithelial Cells

**Figure 2:**
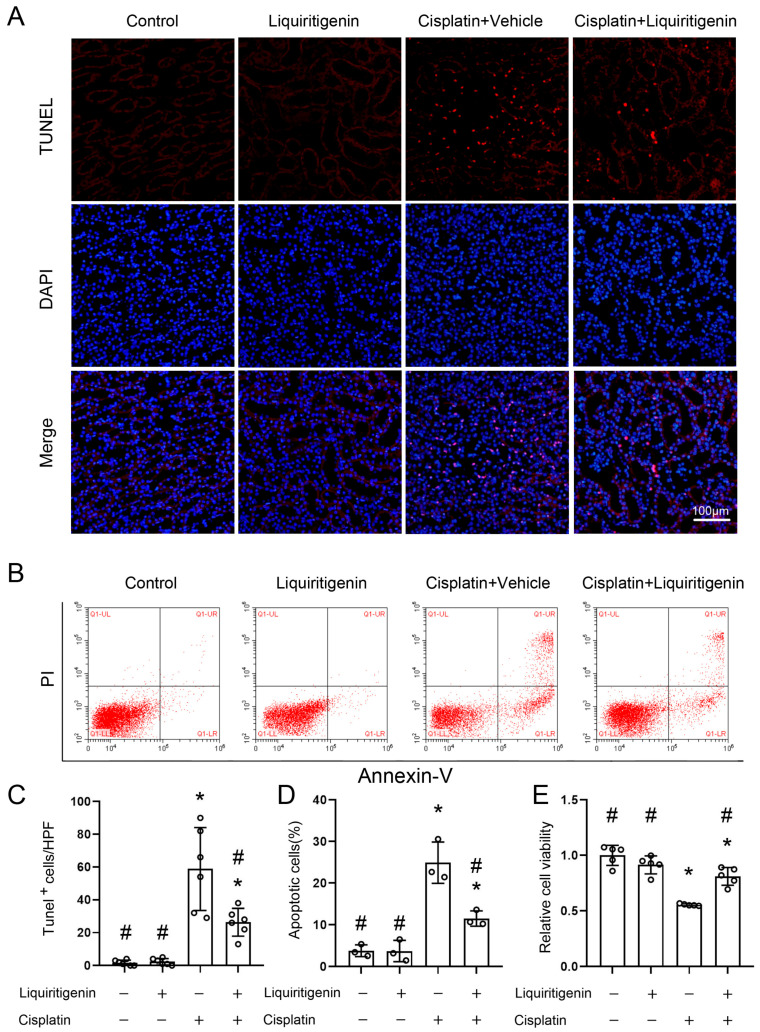
The effect of liquiritigenin on CP-induced apoptosis of renal tubule epithelial cells. (**A**) TdT-mediated dUTP nickend labeling (TUNEL) assays were performed to assess renal cell death. Nuclei were revealed using 4′,6-diamidino-2-phenylindole staining. (**B**) Representative flow charts showed that cell apoptosis was determined by flow cytometric analysis in renal tubule epithelial cells with different treatments. Cells stained with fluorescein isothiocyanate (FITC)-conjugated AnnexinV and propidiumiodide (PI). (**C**) Quantitative assessment of the TUNEL^+^ cells (numbers per high-power field) in mice kidneys. * *p* < 0.05 vs. vehicle-control group, # *p* < 0.05 vs. CP treatment group (*n* = 6 mice/group). (**D**) Quantification of the percentage of apoptotic cells. * *p* < 0.05 vs. vehicle-control group, # *p* < 0.05 vs. CP treatment group. Data were obtained from three independent experiments. (**E**) 3-(4,5-Dimethyl-2-thiazolyl)-2,5-diphenyl-2H-tetrazolium bromide (MTT) assays were performed to assess cell viability in renal tubule epithelial cells with different treatments. * *p* < 0.05 vs. vehicle-control group, # *p* < 0.05 vs. CP treatment group. Data were from five independent biological replicates.

It is well known that apoptosis of renal tubule epithelial cells is a key feature of the pathogenicity associated with AKI. In this study, cell apoptosis was examined by TdT-mediated dUTP nick-end labeling (TUNEL) staining in vivo (Figure 2A,C) and by flow cytometry with Annexin V-FITC and propidium iodide (PI) staining in vitro (Figure 2B,D). Liquiritigenin was found to substantially decrease the number of apoptotic renal tubule epithelial cells compared with the CP treatment group. The result was further confirmed with cell viability rates by MTT cell viability assay (Figure 2E).

### 2.3. Liquiritigenin Attenuated CP-Induced Mitochondrial Damage

**Figure 3:**
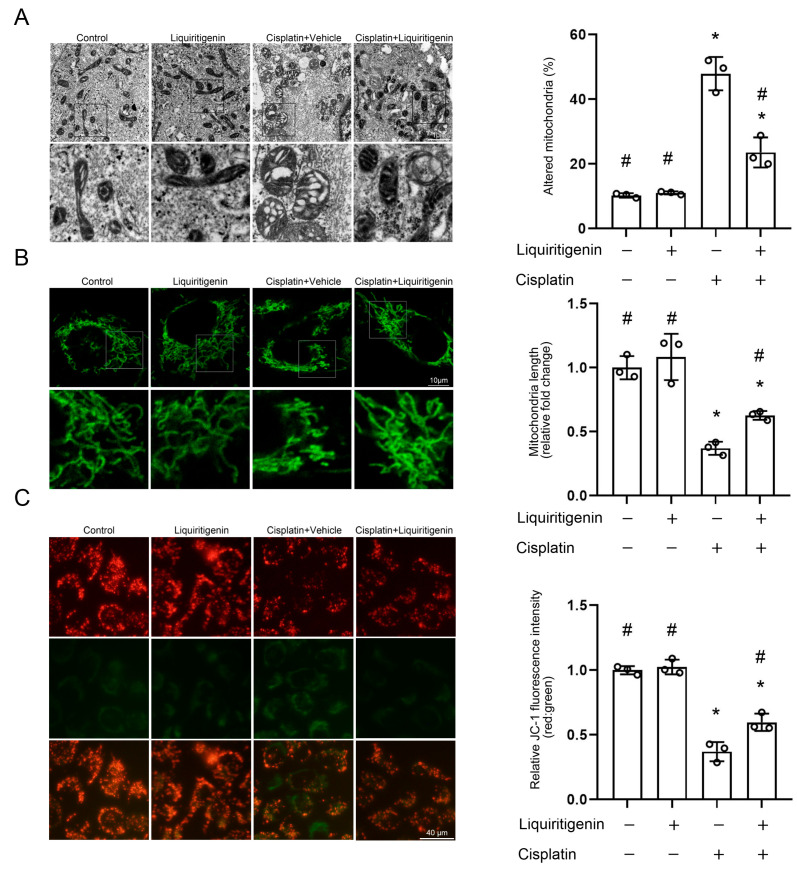
The effect of liquiritigenin on CP-induced mitochondrial damage. (**A**) Representative TEM images on the left showing mitochondrial morphology and mitochondria diameter of live cultured renal tubule epithelial cells with different treatments. Quantitative assessment of the percentage of altered mitochondria characterized by mitochondria swelling, vacuolization, and cristae fragmentation. * *p* < 0.05 vs. vehicle-control group, # *p* < 0.05 vs. CP treatment group. Data were obtained from three independent biological replicates. (**B**) Representative images for MitoTracker greens staining showing mitochondrial morphology and mitochondria length in live cultured renal tubule epithelial cells with different treatments. The results were normalized to the mitochondrial length of the vehicle-control group. * *p* < 0.05 vs. vehicle-control group, # *p* < 0.05 vs. CP treatment group. Data were obtained from three independent biological replicates. (**C**) Representative images of renal tubule epithelial cells stained with JC-1 showing changes in fluorescence intensity in live cultured renal tubule epithelial cells with different treatments. JC-1 fluorescence was normalized to the red-to-green ratio of the vehicle-control group. * *p* < 0.05 vs. vehicle-control group, # *p* < 0.05 vs. CP treatment group. Data were obtained from three independent biological replicates.

In vitro, the cellular amount of fragmented mitochondria was found to increase after CP challenge in renal tubule epithelial cells. In contrast, liquiritigenin was found to reduce CP-induced mitochondrial abnormalities by maintaining the mitochondrial morphology and diameter in renal tubule epithelial cells (Figure 3A). Then, Mito-Tracker Green and JC-1 probes were used to further define the mitochondrial damage in the process. Liquiritigenin treatment significantly inhibited CP-induced mitochondrial fission (Figure 3B). Meanwhile, liquiritigenin effectively attenuated the CP-induced decline in mitochondrial membrane potential (MMP, Figure 3C).

### 2.4. Liquiritigenin Was Predicted as an Adjustor in NRF2-Signaling by Molecular Docking Models

**Figure 4:**
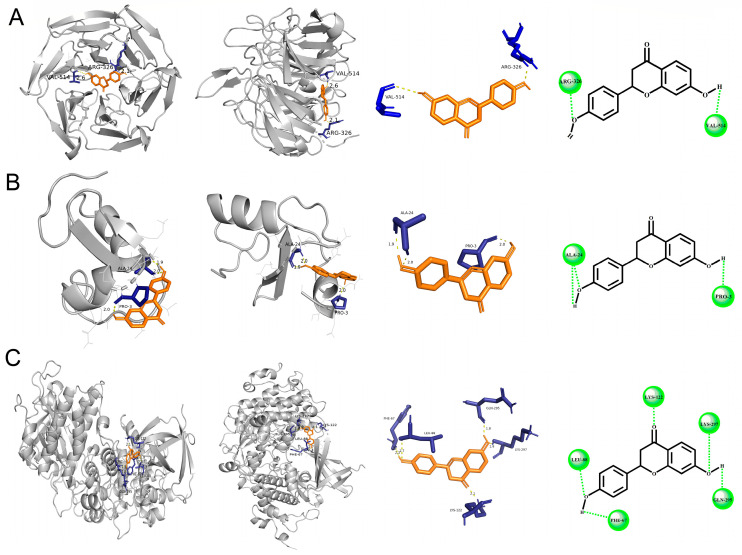
Structure charts of molecular docking models. Docking simulation for the interaction between liquiritigenin with KEAP1 (**A**), HRD1 (**B**), and GSK-3β (**C**) in a general overview, a local overview and 2D overview.

In order to determine the detail mechanisms of the protective effect of liquiritigenin, some receptors in the antioxidant stress pathway as potential binding ligands and the key proteins in molecular docking models were selected. Interestingly, KEAP1, GSK-3β and HRD1, which are regulators of NRF2, all showed binding energies with liquiritigenin (Table 1). As shown in Figure 4, liquiritigenin has potential binding activities to these three molecules in multiple sites.

### 2.5. Liquiritigenin Promoted the Nuclear Translocation of NRF2

**Figure 5:**
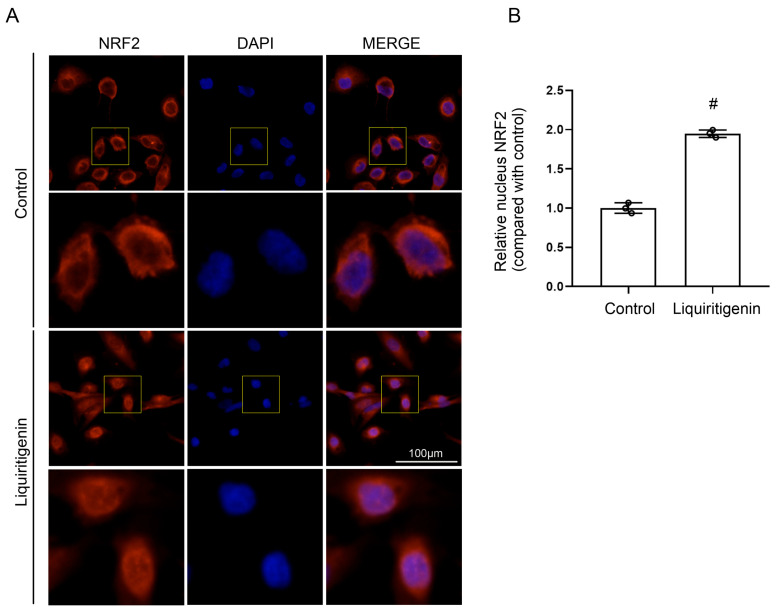
The effect of liquiritigenin on the nuclear translocation of NRF2. (**A**) Subcellular location of NRF2 determined by immunofluorescence microscopy in live cultured renal tubule epithelial cells with liquiritigenin treatment. Nuclei were revealed using 4′,6-diamidino-2-phenylindole staining (DAPI). (**B**) Quantification of nucleus NRF2 fluorescence density. The results were normalized to the ratio of the nucleus NRF2 of vehicle-control. # *p* < 0.05 vs. the vehicle-control group. Data were obtained from three independent biological replicates.

In the cytoplasm, KEAP1, GSK-3β and HRD1 mediate the retrotranslocation and ubiquitination of NRF2. The nuclear translocation of NRF2 is closely related to its activity. Based on the results of molecular docking simulation, fluorescence staining of NRF2 (red) in the live cultured renal tubule epithelial cells was performed to examine whether liquiritigenin can affect the nuclear translocation of NRF2. As shown in Figure 5, liquiritigenin at a concentration of 50 μM could significantly promote nuclear translocation in live cultured renal tubule epithelial cells (Figure 5).

### 2.6. Liquiritigenin Positively Regulated SIRT3 Signaling

**Figure 6:**
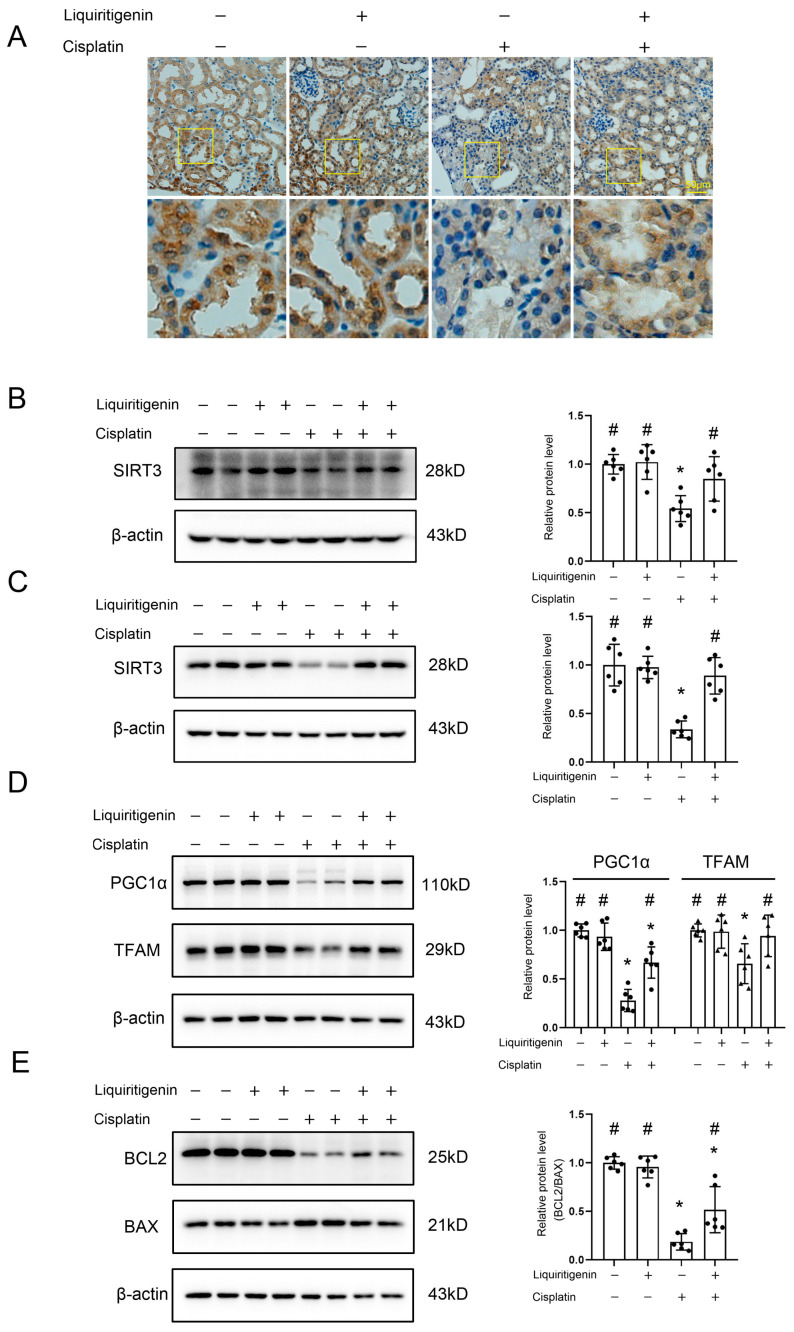
The effect of liquiritigenin on the protein levels through NRF2-SIRT3 signaling. (**A**) Representative photomicrographs of SIRT3 immunohistochemical staining in the kidneys from different groups of mice. (**B**) Representative Western blot gel documents and summarizes the data showing the levels of SIRT3 in the kidney from different groups of mice (*n* = 6 mice/group). * *p* < 0.05 vs. control group of mice, # *p* < 0.05 vs. mice with CP treatment. (**C**) Representative Western blot gel documents and summarized data show the levels of SIRT3 in live cultured renal tubule epithelial cells with different treatments. * *p* < 0.05 vs. control group, # *p* < 0.05 vs. CP treatment group. Data were obtained from six independent biological replicates. (**D**) Representative western blot gel documents and summarized data showing the levels of PGC-1α and TFAM in live cultured renal tubule epithelial cells with different treatments. * *p* < 0.05 vs. control group, # *p* < 0.05 vs. CP treatment group. Data were from six independent biological replicates. (**E**) Representative Western blot gel documents and summarized data showing the levels of BCL2 and BAX in live cultured renal tubule epithelial cells with different treatments. * *p* < 0.05 vs. control group, # *p* < 0.05 vs. CP treatment group. Data were obtained from six independent biological replicates.

Finally, to confirm the effects and mechanisms of liquiritigenin in recovering mitochondrial injury after AKI, the role of liquiritigenin in mitochondrial biogenesis and apoptosis was investigated by detecting expression levels of related proteins in NRF2/SIRT3 signaling. IHC staining revealed that the expression of protein SIRT3 was significantly reduced in the kidneys of mice after CP treatment, particularly in damaged renal tubule cells, while the decreased expression could be restored by liquiritigenin (Figure 6A). Western blot assay further confirmed that liquiritigenin reversed the decreased protein level of SIRT3, which was induced by CP, both in the kidneys of mice (Figure 6B) and in live cultured renal tubule epithelial cells (Figure 6C). The protein levels of PGC1α (Figure 6D), which is the master regulator of mitochondrial biogenesis, and TFAM (Figure 6D), which is involved in mitochondrial DNA replication/translation, were both enhanced by liquiritigenin. In addition, liquiritigenin was found to increase the expression of BCL-2 and reduced protein BAX levels (Figure 6E) in live cultured renal tubule epithelial cells compared to CP treatment, indicating the down-regulation of enhanced cell apoptosis. Together, these results indicated that SIRT3 signaling is essential for liquiritigenin treatment and plays a role in renal protection and mitochondrial dysfunction restoration after AKI.

## 3. Discussion

Deterioration of renal function can be triggered by the nephrotoxicity of many therapeutic drugs, including CP, an important drug that causes AKI. In the progression of CP-induced AKI, the events of impaired mitochondrial function and excessive reactive oxygen species (ROS) production occur, which in turn can disrupt the intracellular redox balance and induce much more serious oxidative stress and mitochondrial dysfunction [23]. Given these mechanisms, the discovery of antioxidants against CP-induced AKI has become significant for the development of treatment strategies. Recent studies have reported that liquiritigenin is a natural antioxidant in arsenic trioxide-induced liver injury and against natural or chemical toxicities [24], and that it attenuates high-glucose-induced mesangial matrix accumulation, and inflammatory and oxidative stress [25]. The current study verified that liquiritigenin could reduce SCr and BUN levels in a CP-induced AKI mice model. In an animal model, the restoration of tubule necrosis, dilatation and damage scores were clearly seen after liquiritigenin treatment. It is well-known that CP easily accumulates in renal tubule epithelial cells of the kidney. Therefore, in this study, renal tubule epithelial cells were chosen as research tools. TUNEL staining, FCM and MTT detection provided clear evidence that liquiritigenin could reduce the apoptosis of renal tubule cells in vitro and in vivo. It was shown that, in renal tubule epithelial cells, liquiritigenin can reduce CP-induced mitochondrial abnormalities by maintaining mitochondrial morphology and mitochondrial membrane potential (MMP). The above results show a significant protective effect of liquiritigenin against CP in AKI.

A recent study found that NRF2-knockout mice are more sensitive to CP and more likely to suffer severe kidney damage than other mice [26]. Furthermore, based on the close association of oxidative stress and mitochondrial dysfunction, this study hypothesized that the protective effect of liquiritigenin depends on the classic antioxidant signaling-NRF2 pathway. Molecular docking models were used to predict the binding activities of liquiritigenin to KEAP1, GSK-3β and HRD1, which are responsible for regulating the activity of NRF2. Results showed that liquiritigenin has potential binding activities to these three proteins in multiple sites. Then, the increased transportation to the nucleus of NRF2 was observed in renal tubule epithelial cells, which implies increased antioxidant activity. Although the present study did not provide conclusive evidence whether NRF2 activation is mediated by the direct inhibition of KEAP1-dependent and non-KEAP1-dependent pathways with liquiritigenin treatment, it can also confirm the role of liquiritigenin in the positive induction of NRF2 activity. Further studies will be planned to investigate the explicit regulation mechanisms underlying NRF2 activation.

As reported previously, NRF2 plays an important role in regulating mitochondrial homeostasis and maintaining mitochondrial function [27]. Some protein levels related to mitochondrial function in mice and cell models were subsequently detected in the current study. NRF2 has been proven to be a direct regulator of SIRT3 against reticulum stress in liver injury [28]. SIRT3, a mitochondrial histone deacetylation enzyme, is primarily localized in mitochondria. It can directly deacetylate and activate the major mitochondrial antioxidant enzymes and mitochondrial metabolic enzymes downstream [29], as well as apoptosis-related proteins [30]. Burgeoning studies have reported that SIRT3 can alleviate CP-induced renal tubule epithelial cell injury by maintaining mitochondrial integrity, further suggesting that improving mitochondrial dynamics by increasing SIRT3 expression has become a potential therapeutic strategy to alleviate CP-induced renal injury [31]. In CP-induced AKI mice and cell models, protein levels of SIRT3 were found to be increased significantly with liquiritigenin treatment, indicating the regulatory ability of liquiritigenin in mitochondrial biogenesis through the NRF2/SIRT3 pathway against CP-induced AKI. To clarify the mechanisms in detail, the expression changes of up- and down-stream proteins of SIRT3, including PGC-1α and TFAM, were detected. PGC-1α is a nuclear-encoded transcription coactivator that regulates the expression of a variety of nuclear-encoded mitochondrial proteins, including mitochondrial antioxidant genes and biogenic genes [32]. TFAM is a member of the high mobility group protein superfamily with the function of stabilizing and maintaining mitochondrial DNA. Meanwhile, protein expression levels of BCL-2 and BAX were detected in the current study, which can be regulated by SIRT3 as the ratio of BCL-2/Bax was decreased in SIRT3 deficiency groups [33]. BAX was found to be a proapoptotic BCL-2 family protein that induces mitochondrial Ca^2+^ overload and the subsequent activation of downstream cell apoptotic events [34]. The inhibition or modulation of BAX, which can block both mitochondrial dysfunction and cell apoptosis, was shown to be important in drug toxicity therapy [34,35]. This study showed that protein levels of SIRT3, TFAM, PGC-1α and BCL-2 were all upregulated while BAX expression was inhibited with liquiritigenin treatment in a CP-induced AKI model. The results described above indicate that the protective effects of liquiritigenin, including the promotion of mitochondrial biogenesis and the inhibition of apoptosis, are related to SIRT3 activity against CP-induced AKI. One recent study has shown that NRF2 could reverse ER stress injury by directly binding to the SIRT3 ARE site [28]. The other study verified that SIRT3 can also play an anti-oxidative stress role by activating the NRF2-signaling pathway [36]. Therefore, it should be noted that the regulation of SIRT3/NRF2 in terms of the mitochondrial function may be a complex feedback regulation and therefore is not monolithic. Even so, based on the current study, it is known for sure that liquiritigenin can affect the mitochondrial function and dysfunction in the NRF2/SIRT3 pathway.

## 4. Materials and Methods

### 4.1. Reagents and Materials

Standard liquiritigenin of purity >98% was purchased from Aladdin Holdings Group Co., Ltd. (Shanghai, China). CP of over 98.5% purity (by HPLC) and the TUNEL kit were purchased from Beyotime Biotechnology (Shanghai, China). Annexin V-FITC/PI double staining apoptosis detection kit were obtained from BestBio (Shanghai, China). The antibodies used for Western blot analysis were as follows: anti-PGC-1α (Mouse, 1:1000, Catalog No.66369-1-Ig), anti-TFAM (Rabbit, 1:1000, Catalog No.22586-1-AP), anti-BCL-2 (Rabbit, 1:1000, Catalog No.26593-1-AP), anti-BAX (Mouse, 1:1000, Catalog No.60267-1-Ig), anti-β-actin (Mouse, 1:6000, Catalog No.66009-1-Ig), purchased from Protein Tech Group (Chicago, IL, USA), and anti-SIRT3 (Rabbit, 1:1000, Catalog No.ab189860), purchased from Abcam (Abcam, Cambridge, UK). Reagents related to cell culture such as culture medium, fetal bovine serum, and streptomycin and penicillin were purchased from Gibco (Shanghai, China).

### 4.2. Experimental Animals

Male BALB/c mice 6~8 weeks, weighing 18~22 g, were obtained from Jinan Pengyue Experimental Animal Breeding Co., Ltd. (Jinan, China), and were housed in cages, with 5 mice in each cage, with free access to food and water in a room with controlled temperature of 20~24 °C and a 12 h light/dark cycle. All animal experiments were conducted in accordance with the Guide for the Care and Use of Laboratory Animals as adopted and promulgated by the U.S. National Institutes for Health, and were approved by the Institutional Animal Care and Use Committee Shandong University (No.ECSBMSSDU2019-2-024). After an adaptive phase of 5 days, mice were randomized into four groups administered with control solvent (0.5% DMSO in 0.9% NaCl injection), CP solution (20 mg/kg body weight/day), liquiritigenin solution (15 mg/kg body weight/day) and liquiritigenin and CP solution by intraperitoneal injection. Liquiritigenin was prepared into stocking solution (200×, 300 mg/mL) with DMSO and then diluted to the concentration 1.5 mg/mL with 0.9% NaCl injection. CP was administrated once on the first day. Liquiritigenin was administrated to CP-induced AKI mice model once/day for a continuous 2 days, and the first injection was at 30 min prior to CP treatment. The injection dose of all solutions was 0.1 mL/10 g. Then, mice were humanitarian executed after 72 h post-injection of CP solution. Blood samples were taken for monitoring renal function and kidney tissues were fixed with formalin [23,37,38,39,40].

### 4.3. Hamatoxylin and Eosin (H&E) Staining

Four-micrometer mice kidney tissue sections were stained with an H&E reagent after dewaxing and rehydrating in the program as described [41]. Pathological section images were obtained using a Ti2-A fluorescence microscope (NIKON, Tokyo, Japan).

### 4.4. Tubular Damage Scores

Under a high-power microscope, 10 fields of renal cortex and outer medulla were randomly selected for observation. Renal morphology was scored as described [38,42].

### 4.5. Immunofluorescence Staining

Renal tubule epithelial cells were treated with liquiritigenin (50 µM) for 6 h, and then washed with phosphate-buffered saline (PBS, pH = 7.2). Immunofluorescent staining was performed as described [41]. The images were visualized under a Ti2-A fluorescence microscope (NIKON, Tokyo, Japan). The antibody used for immunofluorescence staining was anti-NRF2 antibody (1:100, Proteintech, Chicago, IL, USA). Quantitative data from at least 30 cells were counted per group from representative triplicate experiments.

### 4.6. Transmission Electron Microscopy (TEM)

The freshly harvested renal tubule epithelial cell samples were obtained through low-speed centrifugation, and then the supernatant was removed and fixed with glutaraldehyde at 4 ℃. The sample handling and detection were performed by the electron microscopic core Lab of Shandong University as described [43]. Three independent samples were selected from each group and 10 electron micrographs were taken in each sample to analyze the ratio of abnormal mitochondria in the cultured renal tubule epithelial cells.

### 4.7. TUNEL Assay

Sectioning of the paraffin-embedded kidney tissue after dewaxing and rehydrating as shown above were performed with a TUNEL assay following the manufacturer’s protocols (Roche Diagnostics, Mannheim, Germany) as described [41]. The images were visualized under a Ti2-A fluorescence microscope (NIKON, Tokyo, Japan).

### 4.8. Cell Culture and Treatment

Renal tubule epithelial cells (HK-2) were obtained from National Collection of Authenticated Cell Cultures (Shanghai, China) and cultured in complete DMEM-F12 medium with 10% fetal bovine serum and 1% streptomycin and penicillin, at 37 °C with 5% CO_2_ in a humidified incubator [44]. Cells were subcultured every 2~3 days when 80%~90% confluence was reached.

### 4.9. Cell Viability Assay (MTT)

A 200 µL solution of renal tubule epithelial cells was seeded in 96-well plates with a density of 5 × 10^4^ cells per milliliter. After 18 h, cells were co-incubated with CP (20 μM) and liquiritigenin (25 μM) or incubated with CP (20 μM), liquiritigenin (25 μM) separately, for 24 h. The control solvent was complete DMEM-F12 medium with 0.1% DMSO. After incubation, cell viability was measured by MTT assay as described [45]. The OD values of the microplate were measured by a microplate reader (Molecular Devices, CA, USA).

### 4.10. Cell Apoptosis Detection

A 2 µL solution of renal tubule epithelial cells was seeded in a six-well plate with a density of 5 × 10^4^ cells per milliliter. After 18 h, cells were co-incubated with CP (20 μM) and liquiritigenin (25 μM) or incubated with CP (20 μM) and liquiritigenin (25 μM) separately, for 24 h. The control solvent was complete DMEM-F12 medium with 0.1% DMSO. Subsequently, cells were collected and detected by Annexin V-FITC/PI double staining apoptosis detection kit according to the reference manual. Subsequently, 400 μL of binding buffer was added to each sample and then examined using a flow cytometer (Beckman Coulter, Brea, CA, USA).

### 4.11. MitoTracker Green Staining

Renal tubule epithelial cells were incubated with the Mito-Tracker Green (Invitrogen/Molecular Probes, Eugene, OR, USA) according to the manufacturer’s instructions and then detected by a LSM780 laser scanning confocal microscope (ZEISS, Jena, Germany) [43].

### 4.12. Mitochondrial Potential Detection

Probe JC-1 staining was used to measure the mitochondrial membrane potential in renal tubule epithelial cells. Post-treatment, medium/JC-1 working solution (1:1) was added to the cell slides in the plate and incubated for 20 min. The staining solution was removed, and then the cells were gently washed twice with JC-1 staining buffer. The pictures were captured by a Ti2-A fluorescence microscope (NIKON, Tokyo, Japan) [43].

### 4.13. Molecular Docking Simulation

The molecular docking study of liquiritigenin was performed by AutodockTools 1.5.6, and the crystal structures of docking molecules were obtained from the Protein Data Bank (https://www.rcsb.org/ (access on 14 October 2021)). The docking results were optimized using Pymol software. The values of CDocker energy were used as evaluation criteria.

### 4.14. Western Blotting

Collected cells or renal tissues were lysed with RIPA lysis buffer (Beyotime Biotechnology, Shanghai, China) on ice. The cell or tissue lysing reagents were collected, centrifuged at 14,000× *g* for 10 min at 4 °C, and mixed with 5 × SDS-PAGE Sample Loading Buffer (Beyotime Biotechnology, Shanghai, China). Proteins were separated by 10~15% SDS-PAGE and then transferred to polyvinylidene fluoride (PVDF) microporous membranes as described [43]. Membranes were subsequently developed with ECL reagent, and chemiluminescent signals were acquired using Tanon 5200 ChemiDoc imager (Shanghai Tianeng Technology Co., Ltd., Shanghai, China).

### 4.15. Immunohistochemical Staining

Immunohistochemical staining was performed as described [43]. Paraffin-embedded kidney tissue sections were stained with anti-SIRT3 antibody (1:100, Abcam, Cambridge, UK) at 4 °C overnight. Graphs were obtained by using a Ti2-A fluorescence microscope (NIKON, Tokyo, Japan).

### 4.16. Statistical Analysis

All data were expressed as the mean ± SD. All results were reported from at least three independent experimental replicates (biological replicates). GraphPad Prism 8.0 software (GraphPad Software Inc., San Diego, CA, USA) and SPSS 19.0 (SPSS Software Inc., San Diego, CA, USA) were used to analyze the data. The significance of the differences in mean values between and within multiple groups was examined by one-way ANOVA with Dunnett’s multiple comparisons tests. The differences were considered statistically significant at *p* < 0.05.

## 5. Conclusions

**Figure 7:**
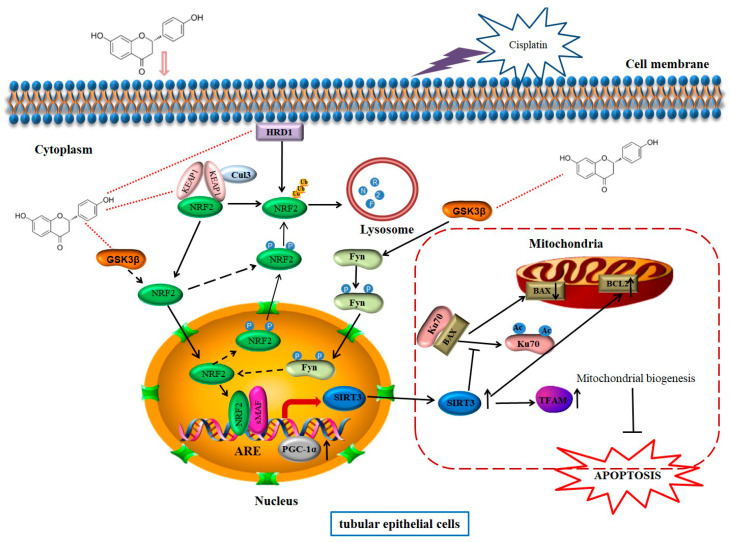
Schematic representation showing the possible mechanisms underlying the protective effect of liquiritigenin against CP-induced AKI. Under pathological conditions, CP leads to mitochondria dysfunction, which contributes to the impaired renal tubule epithelial cells and kidney injury. Firstly, liquiritigenin activates NRF2 by binding to KEAP1, GSK-3β, and HRD1, which can directly inhibit the nuclear translocation and promote the degradation of NRF2 in multiple ways. Subsequently, NRF2 promotes the transcription of SIRT3. In the cytoplasm, SIRT3 deacetylates Ku70, inhibiting the mitochondrial translocation of BAX. Meanwhile, SIRT3 mediates the increased protein level of BCL2. Furthermore, liquiritigenin increases the expression of PGC-1α, which upregulates the SIRT3/TFAM pathway. Eventually, mitochondrial biogenesis is promoted and cell apoptosis is inhibited.

The current study not only showed that liquiritigenin protects against CP-induced AKI by improving mitochondrial function in live cultured renal tubule epithelial cells and BALB/c mice, but also proposed a possible mechanism of liquiritigenin as a potential detoxification drug of CP (Figure 7). This study confirmed that liquiritigenin could be exerted as a nephroprotective agent to protect against CP-induced AKI in a NRF2-dependent manner by improving mitochondria function. The present results provide insights into the underlying molecular mechanisms and will contribute to the development of potential therapeutic natural medicine for CP toxicity.

## Figures and Tables

**Table 1:** Docking energies for optimal conformation of liquiritigenin to KEAP1, GSK-3β and HRD1.

Receptors	Binding Sites	CDocker Energy (kcal/mol)
KEAP1	ARG-326, VAL-514	−6.46
HRD1	PRO-3, ALA-24	−5.78
GSK3β	LYS-297, GLN-295, LEU-88, PHE-67, LYS-122	−6.45

## Data Availability

The data that support the findings of this work are available from the corresponding author upon reasonable request.

## Sample Availability

Samples of the compounds are not available from the authors.
